# What makes for sound science?

**DOI:** 10.1186/s12870-017-1139-7

**Published:** 2017-11-10

**Authors:** Fabrizio Costa, Grant Cramer, E. Jean Finnegan

**Affiliations:** 1 Foundazione Edmund Mach, Trento, Italy; 2 University of Nevada, Reno, USA; 3 CSIRO Agriculture and Food, Canberra, Australia

## Abstract

The inclusive threshold policy for publication in BMC journals including BMC Plant Biology means that editorial decisions are largely based on the soundness of the research presented rather than the novelty or potential impact of the work. Here we discuss what is required to ensure that research meets the requirement of scientific soundness.

*BMC Plant Biology* and the other *BCM-*series journals (https://www.biomedcentral.com/p/the-bmc-series-journals) differ in policy from many other journals as they aim to provide a home for all publishable research. The inclusive threshold policy for publication means that editorial decisions are largely based on the soundness of the research presented rather than the novelty or potential impact of the work. The emphasis on scientific soundness (http://blogs.biomedcentral.com/bmcseriesblog/2016/12/05/vital-importance-inclusive/) rather than novelty or impact is important because it means that manuscripts that may be judged to be of low impact due to the nature of the study as well as those reporting negative results or that largely replicate earlier studies, all of which can be difficult to publish elsewhere, are available to the research community. Here we discuss the importance of the soundness of research and provide some basic guidelines to assist authors to determine whether their research is appropriate for submission to BMC Plant Biology.

Prior to a research article being sent out for review, the handling editor will first determine whether the research presented is scientifically valid. To be valid the research must address a question of biological significance using suitable methods and analyses, and must follow community-agreed standards relevant to the research field.

## Experimental design

The methods should be appropriate for the hypothesis being tested and have adequate controls. A key feature of research that is scientifically sound is adequate replication of the data. The results must be reproducible, that is there must be sufficient replication - this means experimental replication not just technical replicates of the same experiment - to provide confidence that the observations are not due to chance.

There are many different ways to design and statistically analyze plant-related experiments. Depending on the nature of the experiment, experimental replication can be achieved by growing and/or treating plants on separate occasions, the use of different alleles of a mutation, multiple independent transgenic lines, or growth of plants in different environments or across several seasons. The replication required also depends on the question being asked. Here are a couple of examples that illustrate this point: the test is for a cold treatment. One has multiple plants within a growth chamber. A scientist will call these individual plants, biological replicates, which indeed they are. However, these are not experimental replicates, they are technical replicates of a single cold treatment. For statistical purposes, we need replication of the cold treatment in the form of multiple growth chambers (at least 3 replicates) or replication in time using the same growth chamber and treatment. However, if the test is for the cold response of different genotypes, then the different plants within the cold chamber (described as biological replicates above) will provide the replication of the genotypes treatment under that specific treatment. A common mistake is to pool the material from individual experimental/biological replicates prior to library preparation and sequencing for transcriptomic experiments, for example. Pooling material at that stage hides any variation between the experimental replicates and therefore cannot be statistically tested for experimental effects. Depending on the experimental design, ANOVA (analysis of variance) may be a more powerful method for testing your hypothesis than a simple t-test.

If on the other hand, the investigator does a study with many observations taken over time, space or both, then replication and ANOVA become obsolete. Such studies can be conducted on-farm, in unmanaged ecosystems, in the rhizosphere of one plant or a community of plants, in a climate chamber with a time series of observations, etc. In these circumstances, treatments may not be applied, but variability of processes is observed while boundary conditions are controlled. The investigator can base design and analysis on widely known analytical tools such as auto- and cross correlation, auto-regressive state-space models, Fourier-based techniques (e.g. spectral and wavelet analysis) and a variety of geo-statistical methods. All these approaches allow for efficient identification of spatial or temporal processes or the diagnosis of symptoms; they do not depend on treatments, nor do they prohibit experimental treatments. Replicates are not necessary either. Proof that observations are not based on chance, but are reflecting a signal, is obtained from their autocovariance structure. These techniques differ from ANOVA because they are not based on uncorrelated or randomness of observations, but rather they are based on variability structure. Variability is not an obstacle but an opportunity. Are data from one year sufficient to publish? Yes, if as many as possible boundary conditions are observed that made the data turn out the way they did. Most observations of ecosystem processes are hard to replicate exactly, but there is no need when using tools such as these that are common in hydrologic sciences, economic time series, climate change, medical sciences, landscape ecology, physical geography among others.

If in doubt, a statistician should be consulted on the experimental design before starting the experiment, otherwise a considerable amount of time, effort and money may be wasted. Don’t forget that just because a result is statistically significant does not imply that it is biologically relevant, so think carefully about the interpretation of your data.

## The use of transgenic plants

Where transgenic organisms are used, we recommend that a preliminary characterization of at least 3 independent primary transgenic lines showing a similar, stable phenotype be provided. A detailed analysis of at least two lines must be presented. This ensures that the phenotype is likely to be due to the transgene per se, rather than some disruption cause by insertion of the transgene, or as a result of tissue culture during the transformation procedure. The ideal controls for such an experiment are transgene-null segregants isolated from self-progeny of a plant hemizygous for the transgene. This may not always be feasible, for example where the plant concerned is self-incompatible, or the generation time is several years. An alternate control is one that has been transformed with an empty vector or with a transgene carrying an inactive/mutant version of the gene concerned. If the phenotype under study is a seed trait or can be affected by seed quality, then it is important that the test and control seed are harvested from plants grown in parallel and are stored under the same conditions.

## Mapping traits

In case of manuscripts related to quantitative genetic studies, including both QTL mapping and GWAS, there are some fundamental requirements to ensure that the data are sound and can be assessed by the reviewers. The authors should provide essential genotyping data, such as marker order and chromosome location. The study should include sufficient individuals to ensure statistical power. For quantitative traits, the phenotyping should be extensively described and possibly performed for at least two years or across multiple environments in a single year. In the material and methods section the authors should detail the methodologies and software used to perform the analysis. The results should be illustrated with high quality figures, providing both QTL interval and LOD profile; for GWAS provide informative Manhattan plot(s). Tables should be also included reporting the most relevant markers associated to the trait of interest. When previously published data is used, state this clearly in the manuscript, providing a reference to the original data and marker information. In this case the authors should clearly distinguished the data already published from the original data presented in the manuscript.

## Omics analyses

In addition to a solid experimental design for the treatments as described above, there are additional factors to consider for large datasets such as transcriptomics, proteomics and metabolomics. Because these data sets contain a large number of data to be tested, there is an increasing probability of getting false positives. To correct for this, a multiple testing correction factor is used. The most common method is to use the Benjamini-Hochberg correction factor for the false discovery rate [1]. After correction, the *p*-values are known as “adjusted *p*-values”. In addition, these datasets should be deposited in a public database appropriate for the type of data presented.

## Molecule nomenclature

Gene, transcripts, proteins and metabolites should be clearly defined and identified so that there is no confusion about the structure being analysed. For example, gene locus identifications are often updated based upon a new assembly of the genome. Consequently there can be a lot of confusion in the literature of what gene sequence was actually studied, creating considerable difficulty for the reader. It is recommended that the latest, most up-to-date molecular identification number or symbol should be reported along with the database used to determine this. In addition, the appropriate taxonomy ID of the species under investigation should be clearly identified when referring to the reference genome. Gene identifications should follow conventional formatting by using the italics format.

## Reference genes

When quantifying genes by qPCR, one should use at least one reference gene that has been validated not to change in the tissue or treatment under investigation. A reference gene that is valid in one tissue or treatment, may not be valid in another tissue or treatment.

Following these guidelines will help your manuscript to pass the quality control step and progress to peer review, the next stage in the review process.
